# Sushi Domain Containing 2 Dysfunction Contributes to Cancer Progression in Patients with Bladder Cancer

**DOI:** 10.7150/jca.97537

**Published:** 2024-08-13

**Authors:** Wei-Ting Kuo, Yi-Chen Lee, Yi-Fang Yang, Ching-Feng Cheng, Ching-Jiunn Tseng, Kuo-Wang Tsai

**Affiliations:** 1 Division of Urology, Department of Surgery, Kaohsiung Veterans General Hospital, Kaohsiung, Taiwan.; 2 Institute of Clinical Medicine, National Yang Ming Chiao Tung University, Taiwan.; 3 Department of Anatomy, School of Medicine, College of Medicine, Kaohsiung Medical University, Kaohsiung, Taiwan.; 4 Department of Medical Education and Research, Kaohsiung Veterans General Hospital, Kaohsiung, Taiwan.; 5 School of Medicine, Tzu Chi University, Hualien, Taiwan.; 6 Department of Pediatrics, Taipei Tzu Chi Hospital, Buddhist Tzu Chi Medical Foundation, New Taipei City, Taiwan.; 7 Department of Research, Taipei Tzu Chi Hospital, Buddhist Tzu Chi Medical Foundation, New Taipei City, Taiwan.; 8 Department of Nursing, Cardinal Tien Junior College of Healthcare and Management, Taiwan.

**Keywords:** SUSD2, cell cycle, bladder cancer

## Abstract

Bladder cancer is the most prevalent type of cancer in Taiwan, and therefore, enhancing the diagnostic sensitivity of biomarkers for early-stage tumors and identifying therapeutic targets to improve patient survival rates are essential. Although Sushi Domain Containing 2 (SUSD2) dysfunction has been identified in several types of human cancer, its biological role in bladder cancer remains unclear. Analysis of The Cancer Genome Atlas revealed significantly higher expression of SUSD2 mRNA in bladder cancer tissues than in adjacent normal tissues. This elevated expression of SUSD2 significantly correlated with pathological stage (*p* = 0.029), pN stage (*p* < 0.001), and pM stage (*p* = 0.047). Univariate analysis revealed that high SUSD2 expression was associated with decreased overall survival (crude hazard ratio = 1.70, 95% confidence interval = 1.13-2.56, *p* = 0.01). Multivariate analysis revealed a significant correlation between high SUSD2 expression and poor survival outcomes (adjusted hazard ratio = 1.53, 95% confidence interval = 1.01-2.31, *p* = 0.043). IHC analysis revealed a significant correlation between elevated SUSD2 protein levels and unfavorable pathological stages (*p* < 0.001). SUSD2 suppression significantly reduced the proliferation, colony formation, and invasion of bladder cancer cells. In addition, cell cycle analysis revealed that SUSD2 knockdown induced G2/M phase arrestin bladder cancer cells. Tumor Immune Estimation Resource analysis indicated that expression of SUSD2 was significantly associated with macrophage infiltration and M2 macrophage polarization in bladder cancer. In addition, miR-383-5p directly targeted the 3'UTR of SUSD2, with its ectopic expression inhibiting the growth and motility of bladder cancer cells. Our study revealed that miR-383-5p/SUSD2 axis dysfunction may contribute to a poor prognosis for bladder cancer by affecting cell growth, metastasis, and the tumor microenvironment.

## Introduction

Bladder cancer is the ninth most prevalent cancer worldwide, with approximately 430,000 new cases diagnosed every year. Bladder cancer is the 13th leading cause of cancer-related mortality 1. It has a male predominance and is the seventh most common cancer among men worldwide 2. This cancer develops because of genetic susceptibility and exposure to external environmental carcinogens or occupational hazards 3, 4. The molecular etiology of bladder cancer in Taiwan substantially differs from that in other regions because Taiwan's industrial communities and unique dietary practices associated with Chinese medicine. Although current therapies have been demonstrated to have promising efficacy, being associated with a 5-year survival rate exceeding 60% 1-3, some patients with bladder cancer experience aggressive or invasive tumor progression that may not be effectively addressed through current treatment modalities. Drug resistance and metastasis are major concerns that often result in patient mortality after treatment failure. Several studies have highlighted the pivotal role of the tumor microenvironment in cancer progression and therapeutic response, particularly with regard to immune checkpoint inhibitors 5-7. Although immune checkpoint inhibitors have been integrated into clinical practice for patients with bladder cancer, they are not effective for all patients 8. Therefore, further research is required to gain deeper insights into the tumorigenesis of bladder cancer and regulation of the tumor microenvironment. Such insights could inform the refinement of treatment strategies and improve patient outcomes.

SUSD2 is a gene that encodes a protein consisting of 822 amino acids with a transmembrane domain and functional domains characteristic of adhesion molecules 9. Studies have indicated that SUSD2 expression may be associated with both favorable and unfavorable cancer prognoses and that its biological function may be either an oncogene or a tumor suppressor, depending on the cancer type and stage 10-14. However, the clinical effect and biological function of SUSD2 in bladder cancer remain unknown. Therefore, further research is required to fully elucidate the mechanism of action of SUSD2 in bladder cancer.

## Materials and Methods

### *In silico* mRNA profile analysis in patients with bladder cancer

In this study, we performed an *in silico* analysis of mRNA profiles, focusing on patients with bladder cancer. We used the Cancer Genome Atlas (TCGA) to obtain gene expression data for SUSD2 and miR-383-5p, along with clinical information from a cohort of patients with bladder cancer. A total of 374 chemotherapy-naive, invasive, high-grade urothelial tumors from 36 tissue source sites were re-reviewed by four expert genitourinary pathologists. These biospecimens were collected from patients diagnosed with muscle-invasive urothelial carcinoma undergoing surgical resection via either transurethral resection or radical cystectomy. No patient had received prior chemotherapy or radiotherapy for their disease. In addition, we examined the expression levels of SUSD2 across various human cancers by using the Tumor Immune Estimation Resource (TIMER) database. We also explored the correlation between SUSD2 expression and immune cell infiltration by using the TIMER database.

### Patients and specimen collection

Bladder cancer specimens were collected from 189 patients, that is, 160 patients with urothelial carcinoma, 16 with squamous cell carcinoma, and 13 with adenocarcinoma. These specimens were obtained from a bladder cancer tissue array (#BL2081a) supplied by US Biomax (Rockville, MD, USA). Pathological grade was determined using the histological standards set by the World Health Organization 15.

### Immunohistochemical staining

A Novolink Max Polymer Detection System (Leica, Newcastle upon Tyne, UK) was used for immunohistochemical (IHC) analysis in this study. The slides were deparaffinized in xylene and rehydrated in graded alcohols. Antigen retrieval was performed by immersing the slides in Tris-ethylenediaminetetraacetic acid (10 mM, pH 9.0) at 125°C for 10 minutes in a pressure boiler. Endogenous peroxidase activity was blocked by incubating the slides with 3% hydrogen peroxide in methanol for 30 minutes. After blocking at room temperature, the primary antibody (anti-SUSD2; #HPA004117, 1:50 dilution) from Sigma-Aldrich (St. Louis, MO, USA) was immediately applied, and the slides were incubated overnight at 4°C. Following washing with phosphate-buffered saline, the slides were incubated with horseradish peroxidase-labeled secondary antibody for 10 minutes at room temperature, and the sections were counterstained with hematoxylin. The pathologists calculated marker scores for staining based on staining intensity (0, no signal; 1, mild; 2, moderate; and 3, strong) and the proportion of positively stained tumor cells in five high-power fields. Finally, the H-score was used to evaluate SUSD2 expression in bladder cancer.

### Cell lines and culture conditions

The human bladder cancer cell lines BFTC-905, T24, and RT4 were obtained from the Bioresource Collection and Research Center in Taiwan. The cell lines were cultured in Dulbecco's modified Eagle's medium supplemented with 10% fetal bovine serum and 1% penicillin-streptomycin-glutamine.

### Small interfering RNA for SUSD2

Small interfering RNA (siRNA) oligonucleotides targeting SUSD2 were synthesized (Sigma-Aldrich). A scrambled oligonucleotide was obtained from Sigma-Aldrich that served as a negative control. BFTC-905 cells were transfected with either siSUSD2 or the control at a final concentration of 10 nM by using Lipofectamine RNAiMAX (Invitrogen, Thermo Fisher Scientific, Waltham, MA, USA). Subsequently, at 24 h after transfection, total RNA was extracted and subjected to reverse transcription-quantitative polymerase chain reaction (RT-qPCR) to evaluate the efficiency of knockdown.

### Cell proliferation and colony formation assays

For cell proliferation assay, 1,000 BFTC-905 cells were seeded onto 96-well plates and transfected with miR-383-5p mimics, si-SUSD2, or a control. Cell viability was determined using the CellTiter-Glo One Solution Assay (Promega, Madison, WI, USA) at 0,1,2,3 and 4 days. For colony formation assay, 8,000 BFTC-905 cells were plated onto a six-well plate and transfected with si-SUSD2, miR-383-5p mimics, or a control. These cells were incubated at 37°C in a CO_2_ incubator until substantial colonies formed, which typically occurred after 2 weeks. Subsequently, the cells were fixed with 10% formaldehyde solution for 2 min at room temperature. After fixation, the plate was stained with 1 mL of crystal violet solution, destained, and air-dried. Crystal violet was dissolved using 1 mL of 10% acetic acid for quantification, and absorbance was measured at 595 nm by using a Multiskan FC microplate reader (Thermo Fisher Scientific).

### Cell invasion and migration assay

The migration and invasion capabilities of BFTC-905 cells were evaluated using Transwell chambers 16. After cells transfected with si-SUSD2, miR-383-5p mimics, or a control were incubated for 24 h, they were resuspended in a serum-free medium and placed into the upper chamber (100 µL) of a Transwell unit. After 24 hours, the cells were stained with crystal violet and examined under a light microscope.

### Ectopic miR-383-5p expression

BFTC-905 cells were seeded at a density of 1 × 10^6^ cells/mL on a 6-cm dish and transfected with 10 nM miR-383-5p mimics or a control (GenDiscovery Biotechnology, Taipei, Taiwan), with Lipofectamine RNAiMAX used as a transfection reagent (Thermo Fisher Scientific, #13778075). After 24 h, the transfected cells were harvested, and their expression levels were assessed using stem-loop real time (RT)-PCR.

### RT-PCR

Total RNA was extracted using TRIzol reagent (Thermo Fisher Scientific, #15596026), then RNA (1 μg) was reverse-transcribed using stem-loop RT-PCR with SuperScript III Reverse Transcriptase (Invitrogen) and oligod T primers for protein-coding gene and miR-383-5p-RT primers for miR-383-5p. Subsequently, SUSD2 expression was examined using the SUSD2-F and SUSD2-R paired primers. The expression of miR-383-5p was evaluated using the primers miR-383-5p-GSF and universal-R, as detailed in a previous study 17. U6 expression was used as an internal control for small RNA, whereas GAPDH was used as an internal control for general gene expression. For gene expression, RT-qPCR was conducted using a SYBR system with the following primer sequences:

SUSD2-F: CAGCTACCAGAGATGGCGAG

SUSD2-R: CCTCTCGGAAGTCATCGCTC

GAPDH-F: 5'-TGCACCACCAACTGCTTAGC-3'

GAPDH-R: 5'-GGCATGGACTGTGGTCATGAG-3'

miR-383-5p-RT: 5'-CTCAACTGGTGTCGTGGAGTCGGCAATTCAGTTGAGAGCCACAA-3'

miR-383-5p-GSF: 5'-CGGCGGAGATCAGAAGGTGATT-3'

Universal reverse: 5'-CTGGTGTCGTGGAGTCGGCAATTC-3'

U6-F: 5'-CTCGCTTCGGCAGCACA-3'

U6-R: 5'-AACGCTTCACGAATTTGCGT-3

### Luciferase reporter assay

The full-length 3'UTR of SUSD2 and a miR-383-5p binding site mutant were cloned into a pMIR-REPORT vector (AM5795, Thermo Fisher Scientific). Subsequently, a pMIR-REPORT-SUSD2-3'UTR or REPORT-SUSD2-3'UTR(mut) vector was respectively cotransfected with or without miR-383-5p mimics into a bladder cancer cell line by using Lipofectamine 2000 (Invitrogen, Thermo Fisher Scientific). After 48 h of transfection, cell lysates were used to measure luciferase activity in a Dual-Glo Luciferase Assay System (Promega).

### Statistical analysis

A chi-square test was conducted to determine the correlation between SUSD2 expression and demographic and clinical variables including age, sex, cancer grade, cancer stage, and pathology. Student's *t* test was conducted to identify significant differences between treatment groups. A *p* value of less than 0.05 was considered to indicate significance.

## Results

### High expression levels of SUSD2 mRNA are associated with a poor prognosis

Analysis of SUSD2 expression across various types of cancer revealed significant upregulation of SUSD2 in bladder urothelial carcinoma, breast invasive carcinoma, cholangiocarcinoma, head and neck squamous cell carcinoma, liver hepatocellular carcinoma, and thyroid carcinoma. By contrast, SUSD2 expression was significantly downregulated in colon adenocarcinoma, kidney chromophobe, kidney renal clear cell carcinoma, kidney renal papillary cell carcinoma, lung adenocarcinoma, lung squamous cell carcinoma, and rectum adenocarcinoma, indicating a dual role of SUSD2 across different types of cancer. Despite these findings, the clinical value and biological role of SUSD2 in bladder cancer remain to be elucidated.

**Figure 2:**
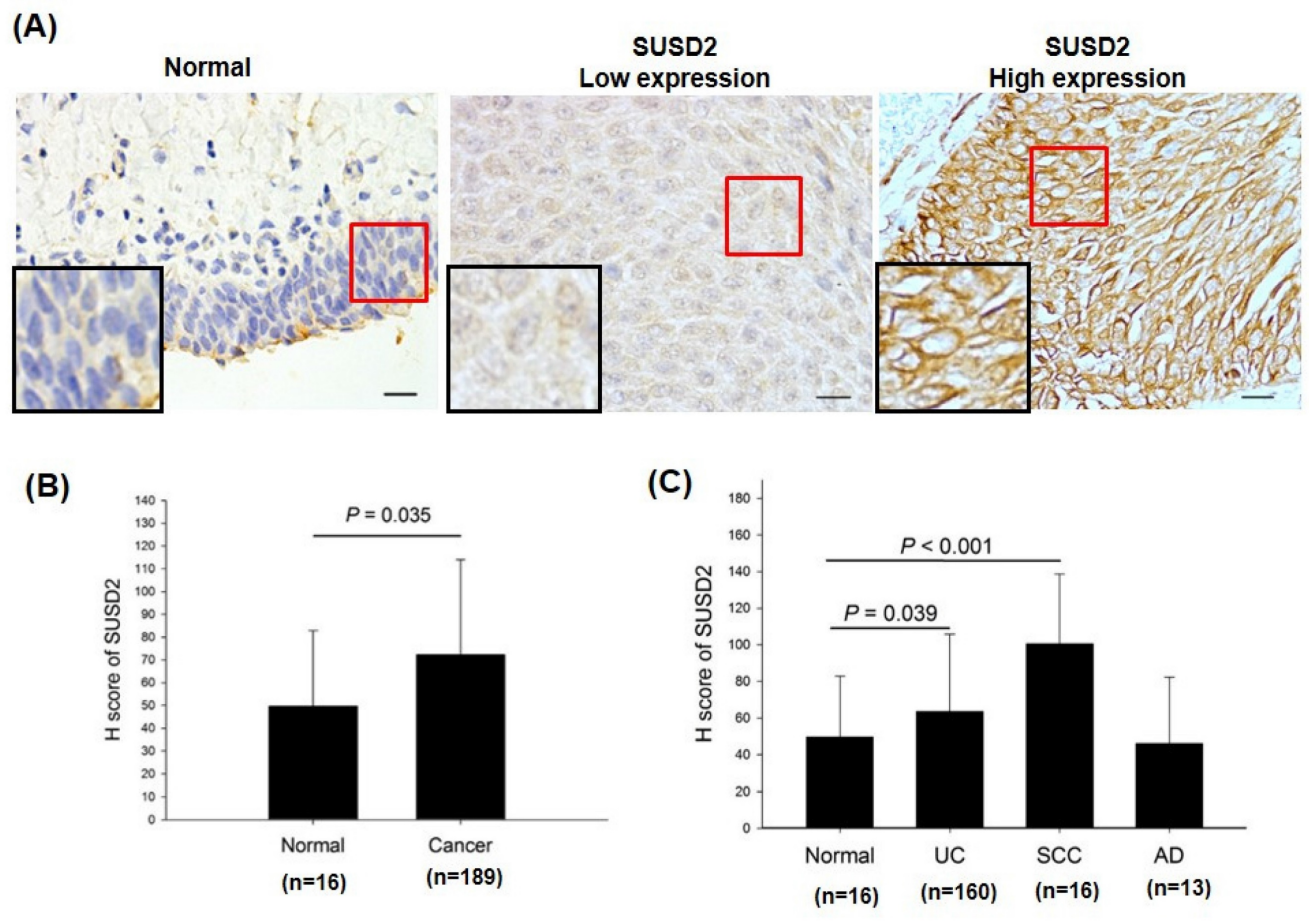
**IHC analysis of SUSD2 protein in bladder cancer.** (A) Expression of SUSD2 in bladder cancer tissues. (B, C) Quantification of SUSD2 expression in patients with bladder cancer. N: normal; UC: urothelial carcinoma; SCC: squamous cell carcinoma; AD: adenocarcinoma. Data are presented as means ± standard deviations; ^*^p < 0.05; ns = not significant.

To determine the clinical effect of SUSD2 on bladder cancer, we analyzed data from TCGA. We analyzed 379 cases to determine the correlation between clinical outcomes and SUSD2 gene expression. The results indicated that the SUSD2 mRNA levels were significantly higher in bladder cancer tissues than in adjacent normal tissues (*p* < 0.001, **Figure 2B**). As presented in **Table 1**, high SUSD2 expression levels significantly correlated with advanced pathological stage (*p* = 0.029), pN stage (*p* < 0.001), and metastasis (*p* = 0.047). We also investigated the correlation between SUSD2 mRNA expression and overall survival (OS) in patients with bladder cancer. By conducting receiver operating characteristic analysis, we established a cutoff value to categorize patients into high and low SUSD2 expression groups. Kaplan-Meier survival analysis revealed that the patients with high SUSD2 expression had shorter survival times than did those with low expression (*p* = 0.01, **Figure 2C**). Multivariate Cox regression analysis revealed that the expression of SUSD2 served as an independent predictor of poor OS (adjusted hazard ratio = 1.53, 95% confidence interval = 1.01-2.31, *p* = 0.006) in the patients with bladder cancer (**Table 2**).

### SUSD2 protein upregulation significantly correlates with unfavorable clinical outcomes in patients with bladder cancer

To determine the protein levels of SUSD2 in patients with bladder cancer, we analyzed the expression of SUSD2 in tumors from 189 patients by using immunohistochemistry and correlated these levels with clinicopathological features. As presented in **Figure 2A**, SUSD2 protein expression was divided into low- and high-expression groups. The expression of SUSD2 was significantly higher in bladder cancer tissues than in normal bladder tissues (*p* = 0.035, **Figure 2B**). In addition, SUSD2 protein expression significantly correlated with advanced pathological stage (*p* < 0.0001) and various pathological subtypes (*p* = 0.002, **Table 3**). Stratification analysis revealed a significant increase in SUSD2 expression in both urothelial carcinoma (*p* = 0.039) and squamous cell carcinoma (*p* < 0.001) relative to that in normal tissues (**Figure 2C**). In summary, SUSD2 upregulation in bladder cancer is associated with advanced pathological stages, indicating a potential role for SUSD2 in the progression or cell motility of bladder cancer.

### SUSD2 knockdown reduces the viability, colony formation, invasion, and migration of bladder cancer cells

**Figure 3:**
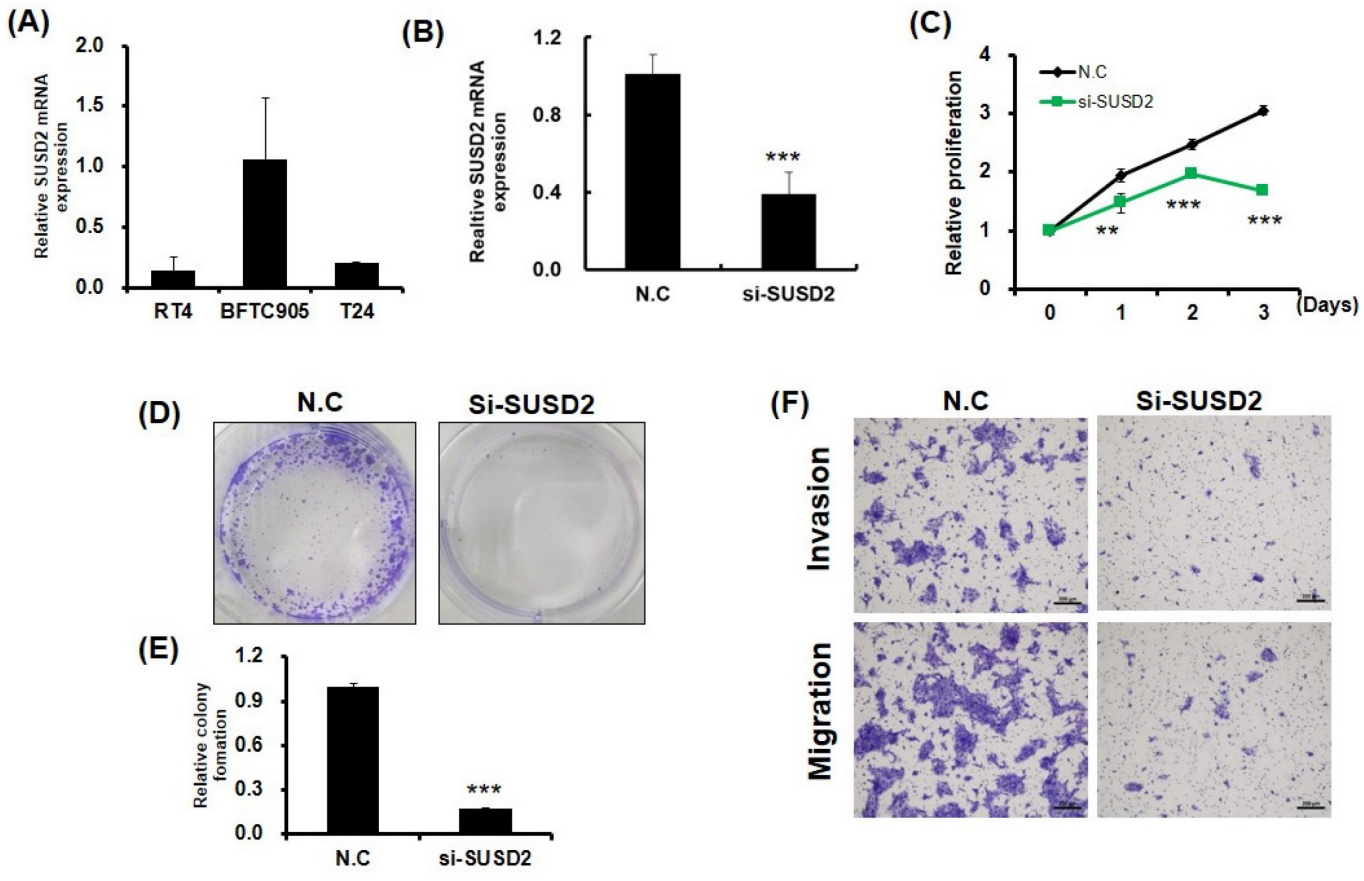
** Reduction in growth, colony formation, migration, and invasion of bladder cancer cells due to SUSD2 downregulation.** (A) SUSD2 expression levels in three bladder cancer cell lines, determined using RT-qPCR. (B) RT-qPCR analysis of SUSD2 expression in BFTC-905 cells after SUSD2 siRNA transfection. (C) Effect of SUSD2 knockdown on viability of BFTC-905 bladder cancer cells. After SUSD2 knockdown, the ability of BFTC-905 cells to proliferate was assessed at 0, 1, 2, and 3 days. (D) Representative images of colony formation assays of BFTC-905 cells with control or SUSD2 siRNA transfection. (E) Colony formation abilities of BFTC-905 cells. Data are presented as means ± standard deviations; ^**^p < 0.001. (F) Migration and invasion capabilities of BFTC-905 cells transfected with SUSD2 siRNA or siRNA control, analyzed using a Transwell assay.

**Figure 4:**
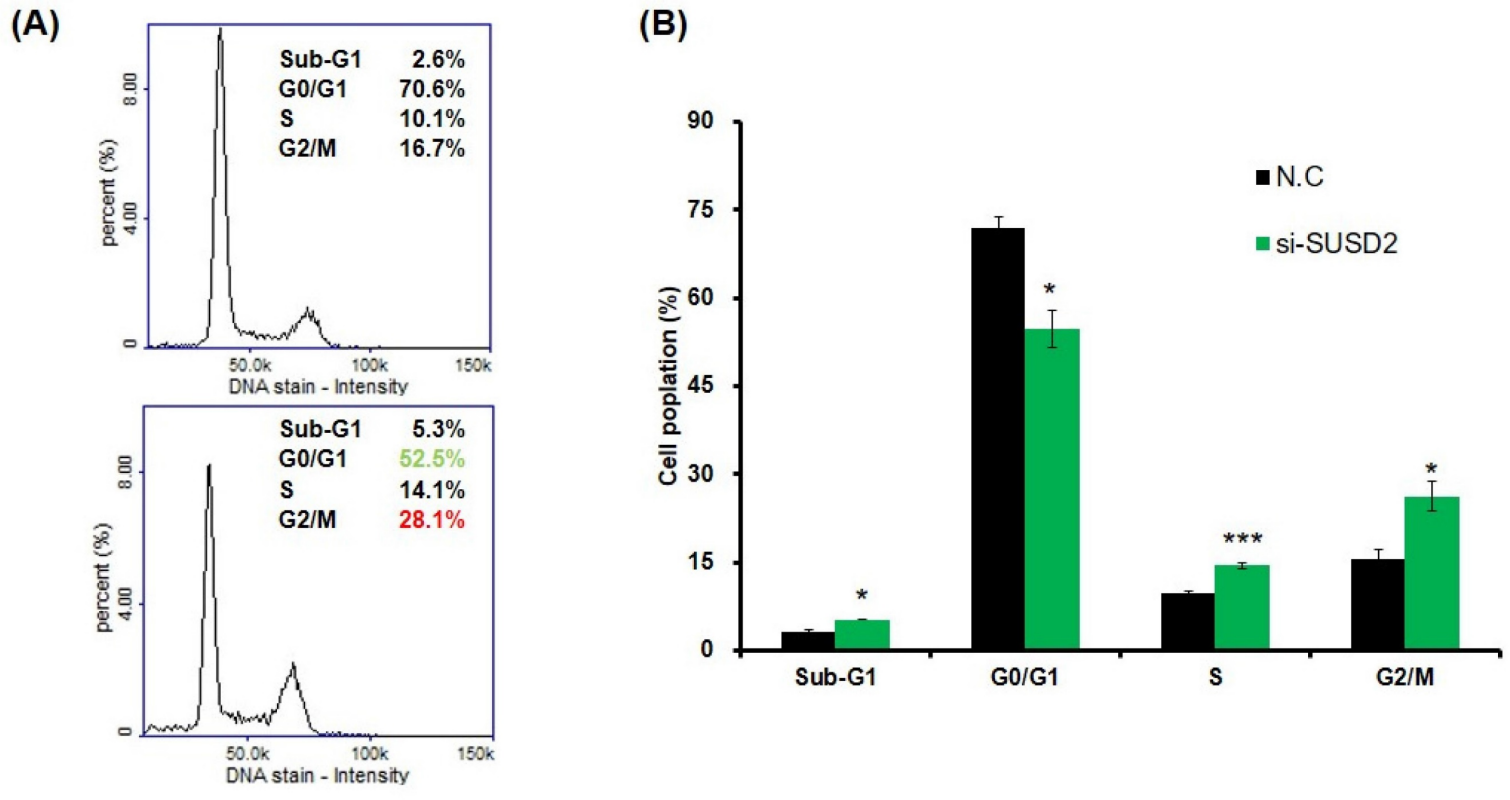
** Impairing cell cycle progression of bladder cancer cells due to SUSD2 downregulation.** (A) Cell cycle analysis of BFTC-905 cells after SUSD2 siRNA or control transfection. (B) Quantification of cell cycle distribution from three independent experiments; ^*^p < 0.05; ^**^p < 0.01; ^***^p < 0.001.

Clinical evidence indicates that SUSD2 is involved in the progression of bladder cancer. To determine the effect of SUSD2 silencing on the migration, invasion, colony formation, and proliferation of bladder cancer cells, we first analyzed SUSD2 expression. As presented in **Figure 3A**, the SUSD2 expression was high in BFTC-905 cells. Therefore, we selected BFTC-905 cells for subsequent biological function assays through a loss-of-function strategy. Our results indicated that the expression of SUSD2 was significantly downregulated in BFTC-905 cells after they were subjected to si-SUSD2 transfection for 48 h (**Figure 3B**). Silencing SUSD2 with siRNA also reduced the cell proliferation (**Figure 3C**), colony formation (**Figures 3D** and **3E**), and migration and invasion (**Figure 3F**) of the BFTC-905 cells relative to those in controls. Analysis of cell cycle phases revealed a significant decrease in the G0/G1 phase population and a significant increase in the sub-G1, S and G2/M phase population in BFTC-905 cells with SUSD2 knockdown, indicating that SUSD2 silencing inhibited bladder cancer cell growth by disrupting cell cycle progression and inducing cell apoptosis (Figure 4A and 4B).

### miR-383-5p silences SUSD2 expression by targeting its 3'UTR

**Figure 5:**
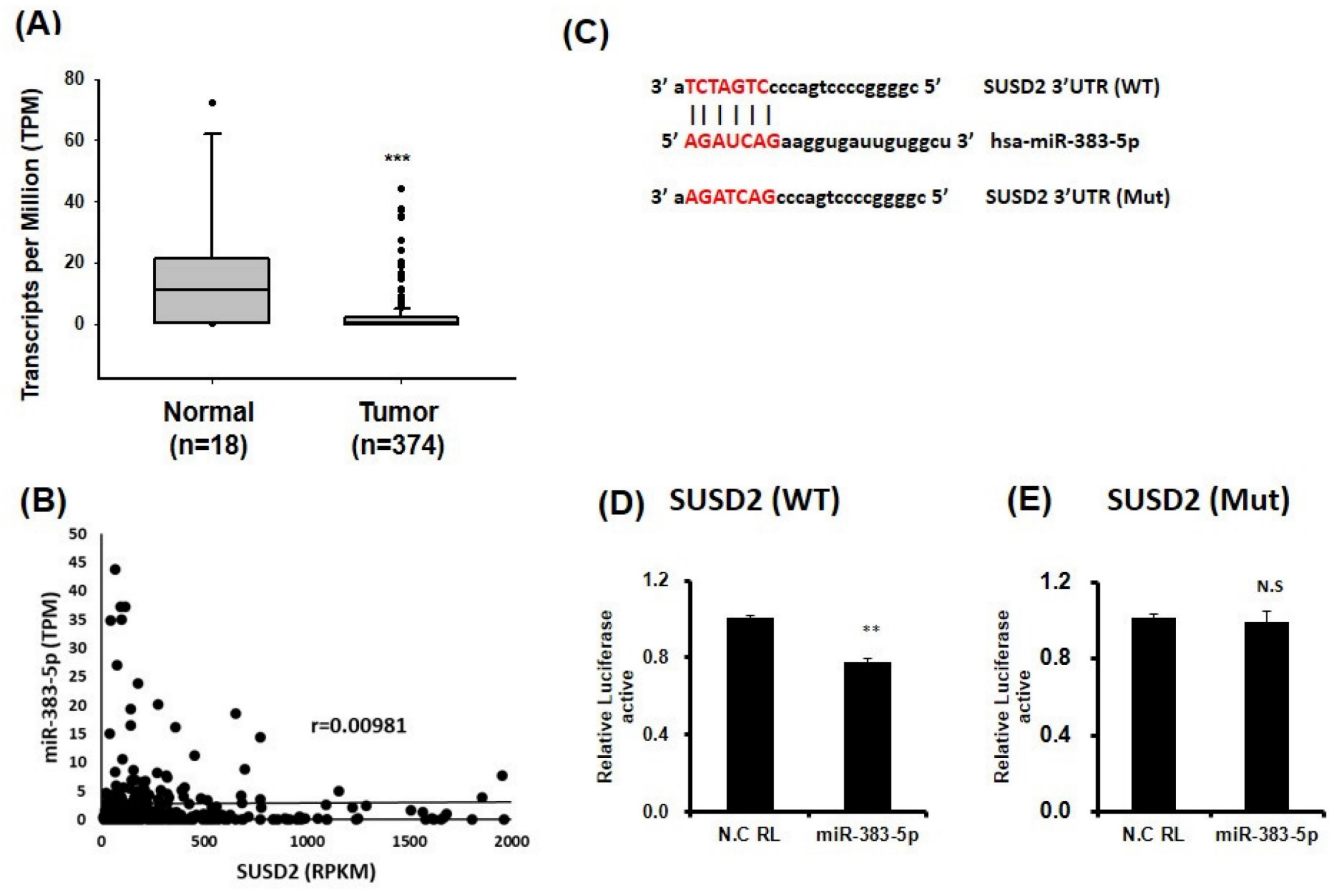
** Direct binding of miR-383-5p to the 3'UTR of SUSD2 in bladder cancer cells.** (A) miR-383-5p expression in bladder cancer cells, analyzed through TCGA. (B) The correlation between miR-383-5p and SUSD2 was analyzed in bladder cancer tissues obtained from the TCGA database. (C) miR-383-5p target sequence within the 3'UTR of SUSD2. Constructs include pMIR-REPORT-SUSD2 (wild type) and pMIR-REPORT-SUSD2 (mutant). (D) and (E) Relative luciferase activity the reporter with the 3'UTR of SUSD2-WT or mutant of SUSD2, determined after cotransfection with miR-383-5p or control.

To determine the molecular mechanism underlying the effect of SUSD2 on cancer cell progression, we used bioinformatics to identify miRNAs and discovered that miR-383-5p targeted the 3'UTR of SUSD2. Analysis of miR-383-5p expression in bladder cancer tissues revealed significant downregulation relative to in normal tissues (**Figure 5A**) by analyzing TCGA database. Further analysis showed that there was no significant negative correlation between miR-383-5p and SUSD2 mRNA expression in bladder cancer tissues **(Figure 5B)**. Luciferase reporter assay analysis confirmed that miR-383-5p directly targeted the 3'UTR of SUSD2, reducing its luciferase activity (**Figure 5C and 5D**), whereas mutation at the SUSD2 binding site could restored this effect (**Figure 5E**).

**Figure 6:**
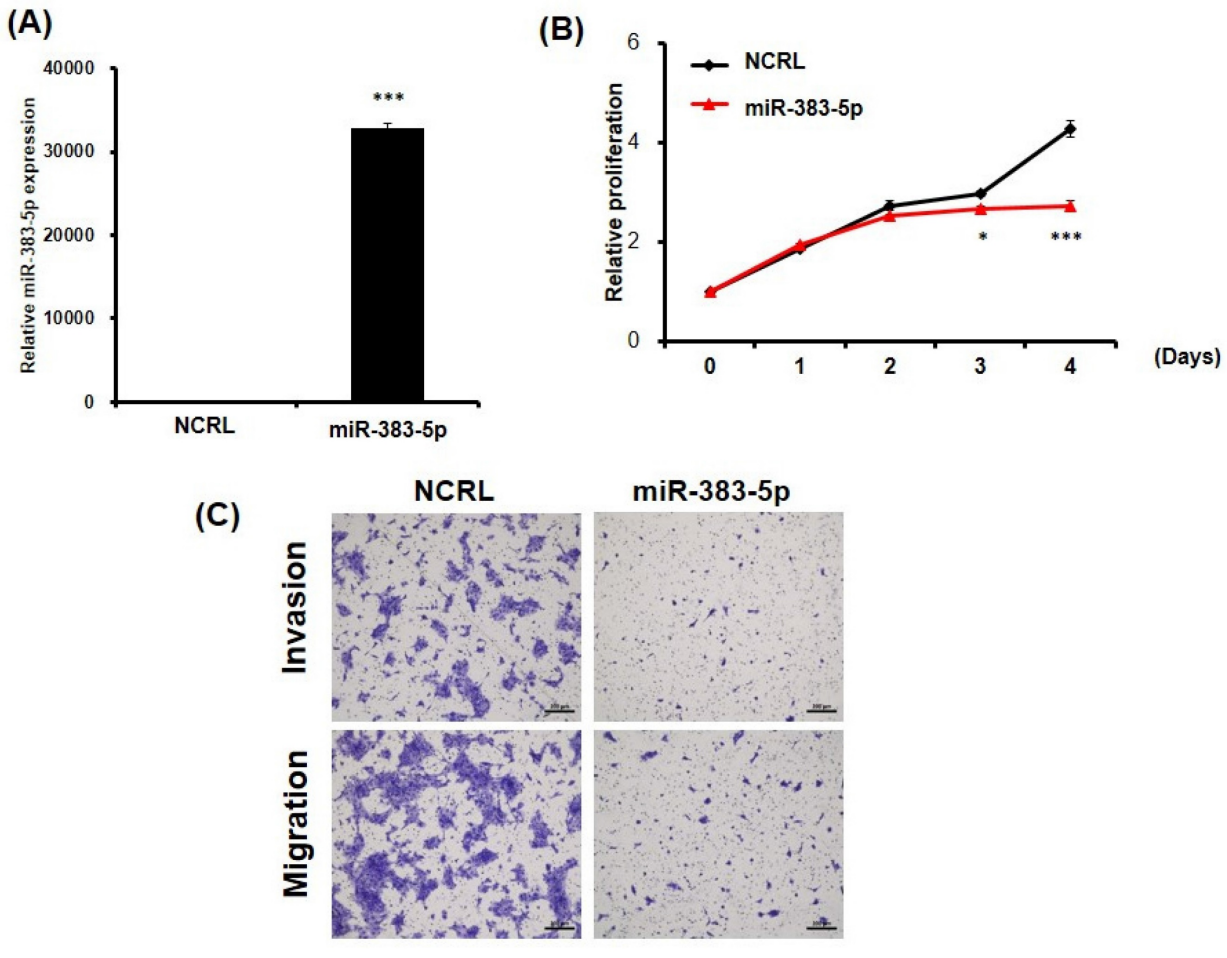
** Potential suppression of growth and motility of bladder cancer cells by ectopic expression of miR-383-5p.** (A) RT-qPCR analysis of miR-383-5p expression in BFTC-905 cells after transfection with miR-383-5p mimics or control. (B) Proliferation of BFTC-905 cells after transfection with miR-383-5p mimics for 0, 1, 2, 3, and 4 days. (C) Migration and invasion capabilities of BFTC-905 cells with miR-383-5p overexpression or control, analyzed using a Transwell assay.

The above results imply that miR-383-5p might suppress SUSD2 expression by blocking the translation process. The ectopic expression of miR-383-5p was similar to that of SUSD2 knockdown in BFTC-905 cells, and transfection with miR-383-5p mimics significantly inhibited the proliferation, migration, and invasion of the BFT-905 cells (**Figures 6A**-**6C**). In summary, dysfunction of the miR-383-5p/SUSD2 axis may contribute to a poor prognosis for bladder cancer because it may affect the growth and metastasis of bladder cancer cells.

### SUSD2 expression correlates with macrophage infiltration in bladder cancer cells

**Figure 7:**
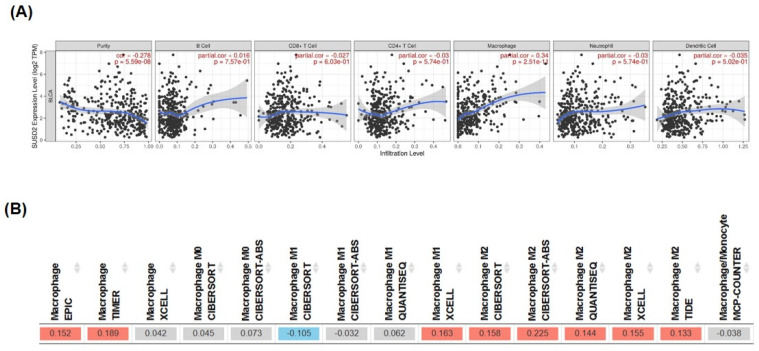
** Significant correlation of high SUSD2 expression with macrophage infiltration and M2 macrophage polarization in bladder cancer.** (A) Correlation between SUSD2 expression and immune cell infiltration, analyzed using the TIMER database. (B) Correlation between SUSD2 expression and M2 macrophage distribution, analyzed using the TIMER database.

TIMER database analysis revealed that the expression of SUSD2 significantly correlated with macrophage infiltration (correlation = 0.34, *p* = 2.51E-11, **Figure 7A**). High SUSD2 expression also positively correlated with M2 macrophage polarization in bladder cancer (**Figure 7B**), indicating involvement of SUSD2 in the tumor microenvironment through macrophage recruitment and M2 polarization. In summary, this study provides novel insights into how the miR-383-5p/SUSD2 axis influences the prognosis of bladder cancer by modulating cancer cell growth, metastasis, and the tumor microenvironment.

## Discussion

SUSD2 plays a complex role in human cancer and exhibits duality influenced by the type of cancer. Extensive research has indicated that SUSD2 often functions as a tumor suppressor in various types of cancer, including ovarian, colon, endometrial, hepatocellular, retinal (retinoblastoma), and lung cancer, inhibiting cancer cell growth, angiogenesis, and metastasis 13, 14, 18-25. In certain types of cancer, such as gastric, breast, and ovarian cancer, elevated SUSD2 expression correlates with increased cancer cell growth and metastasis, contributing to a poor prognosis 14, 20, 24, 25. To the best of our knowledge, our study represents the first comprehensive analysis of SUSD2 in bladder cancer, providing valuable insights for future research. In this study, we used two different cohorts and approaches to evaluate SUSD2 expression. Despite the variations between the cohorts, our data indicated that SUSD2 overexpression was observed in both. This suggests that SUSD2 upregulation in bladder cancer, compared to corresponding adjacent normal tissues, is consistent regardless of cohort differences. We explored the biological function of SUSD2 in bladder cancer, particularly its effects on cell growth and motility. Our findings indicate that silencing SUSD2 significantly reduces bladder cancer cell proliferation and colony formation by disrupting cell cycle progression and motility. Additionally, we presented evidence of a potential link between high SUSD2 expression and advanced disease stage in patients with bladder cancer. In addition to analyzing public databases, we conducted IHC analysis and discovered significant SUSD2 overexpression in bladder cancer, with high levels associated with poor pathological stages. However, the clinical specimens were primarily from Western populations, which may limit the global clinical significance and applicability of our results. Future studies should include bladder cancer specimens from diverse ethnic groups to verify SUSD2 expression levels and address the limitations of this research.

Dysregulated expression of SUSD2 in human cancer can be attributed to various factors, including promoter methylation, genetic variations, dysfunctional oncogenic signaling, and miRNA regulation. Whereas previous studies have associated SUSD2 suppression with promoter hypermethylation 26, we revealed a significant increase in SUSD2 expression in bladder cancer tissue relative to in adjacent normal tissues, indicating other regulatory mechanisms, such as dysregulated signaling or miRNA involvement. Studies have indicated that the Notch3 and TGF-β signaling pathways, which are crucial in bladder cancer progression 27, 28, are implicated in the regulation of SUSD2 expression 12, 29. In addition, several miRNAs, including miR-141-3p, miR-346, and miR-127, have been found to regulate SUSD2 expression through direct interaction 22, 30-32. In this study, we identified miR-383-5p as a miRNA capable of interacting with the 3'UTR of SUSD2 and to thus serve as a tumor suppressor in bladder cancer. Consistent with our findings, those of another study indicate that the expression levels of miR-383-5p are significantly low in bladder cancer, with its ectopic expression capable of suppressing bladder cancer cell growth and metastasis 33. Taken together, our findings indicate that the dysfunction of the miR-383-5p/SUSD2 axis may contribute to the poor prognosis of bladder cancer by affecting cancer cell growth and metastasis. Overall, this study highlights the potential importance of the miR-383-5p/SUSD2 axis in determining the prognosis of bladder cancer by modulating cancer cell growth and metastasis.

Because immune checkpoint inhibitors play a key role in the treatment of bladder cancer 8, understanding the immunosuppressive role of SUSD2 within the tumor microenvironment is essential. According to the literature, SUSD2 interacts with Galectin-1, facilitating tumor immune evasion, angiogenesis, and metastasis in breast cancer 9. It also enhances breast cancer cell invasion and contributes to immune evasion by inducing apoptosis in Jurkat T cells 10. In mouse models, SUSD2 expression is associated with accelerated tumor formation and reduced survival, along with a decrease in CD4 tumor-infiltrating lymphocytes 10. Hultgren et al. 34 reported a significant increase in SUSD2 expression in breast cancer that was strongly correlated with an abundance of M2 macrophages, as identified through IHC analysis. They also discovered that ectopic SUSD2 expression seemed to upregulate the expression of MCP-1, leading to an increase in M0 macrophages polarizing toward the M2 phenotype in breast cancer cells. SUSD2 serves as a negative regulator of antitumor immunity by suppressing the function of CD8+ T cells 18, 35. In mouse models, the absence of SUSD2 (susd2-/-) results in an increase in CD8+ T cells producing antitumor molecules, which mitigate tumor growth. SUSD2 interacts with IL-2 receptor α through a sushi-domain-dependent mechanism. This interaction results in the suppression of IL-2 binding to its receptor, thereby diminishing the IL-2 signaling activation of CD8+ T cells and reducing their antitumor efficacy. According to the literature, SUSD2 expression may protect high-grade serous ovarian cancer cells from immune surveillance by inhibiting platelet aggregation and adhesion to cancer cells, which indicates that SUSD2 plays a dual role in various cancer environments 36. In this study, we observed a significant correlation between high SUSD2 expression and increased macrophage infiltration in bladder cancer, indicating that SUSD2 may play a role in macrophage polarization toward the M2 phenotype, which supports tumor growth, angiogenesis, and metastasis 37.

In conclusion, this study provides novel insights into how the miR-383-5p/SUSD2 axis influences the prognosis of bladder cancer by modulating cancer cell growth, metastasis, and the tumor microenvironment. Although the specific role of SUSD2 in the tumor microenvironment of bladder cancer remains unclear, its influence on macrophage polarization can be considered in the development of innovative therapeutic strategies.

## Figures and Tables

**Figure 1:**
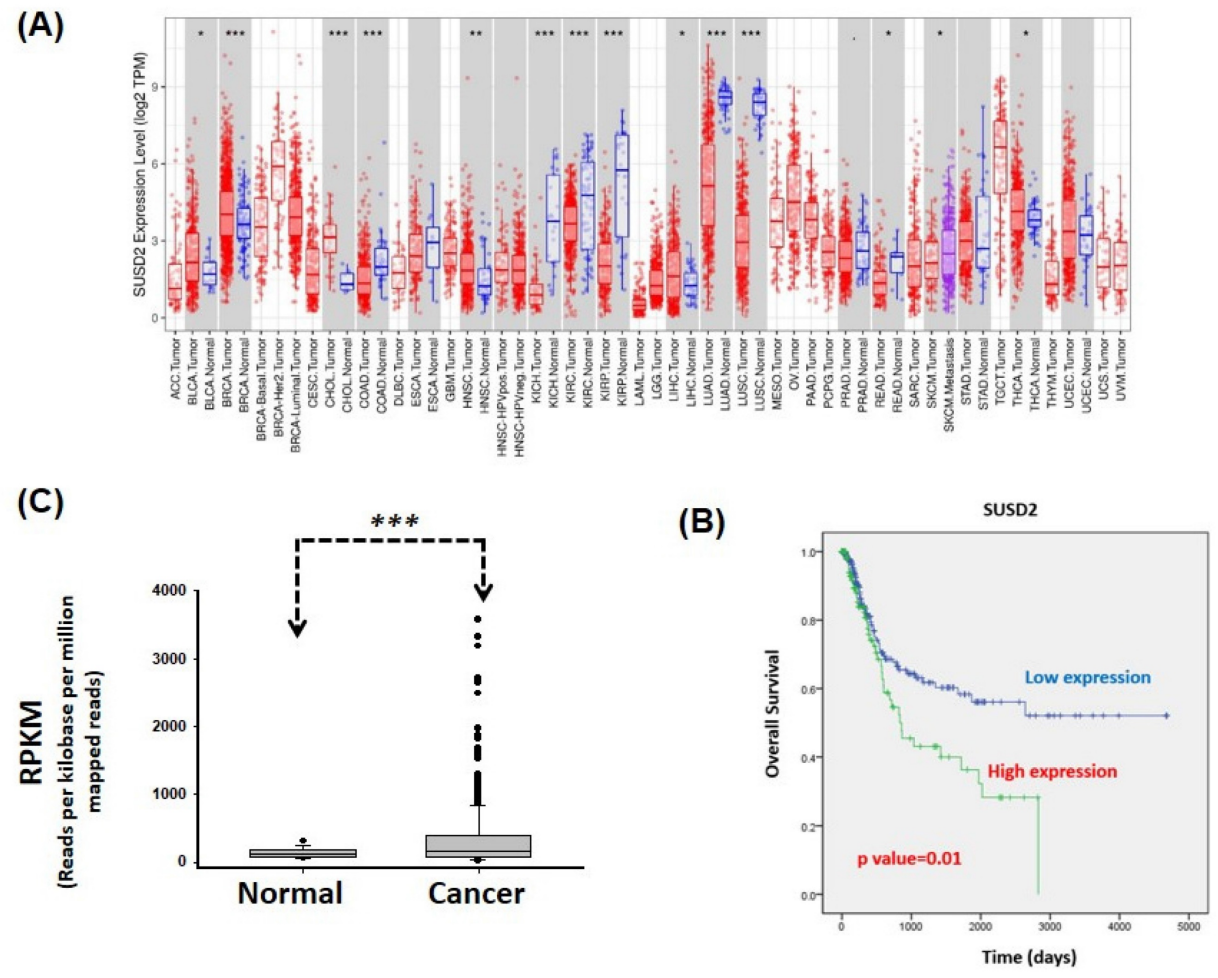
***In silico* analysis of SUSD2 gene expression.** (A) SUSD2 expression levels across various types of human cancer, analyzed using the TIMER database. (B) Relative SUSD2 mRNA levels in bladder cancer tissues, analyzed using TCGA. (C) Correlation between SUSD2 expression and overall survival in patients with bladder cancer, determined using Kaplan-Meier analysis.

**Table 1:** Correlation of SUSD2 expression with clinicopathological characteristics of 374 bladder cancer patients.

Variables	SUSD2 (n=374)			
	No. (%)	Mean±SD	Median	p-value
**Pathology stage**				
I	2 (0.5)	213.45±233.62	213.45	0.046^a^
II	109 (29.1)	238.82±256.66^d^	147.02	
III	134 (35.8)	298.17±411.67^e^	155.21	
IV	129 (34.5)	545.44±928.46^de^	236.32	
**pT stage** (n=372)				
T1	3 (0.8)	199.81±166.88	172.52	0.152^a^
T2	124 (33.3)	264.48±409.09	139.71	
T3	190 (51.1)	403.58±728.24	188.92	
T4	55 (14.8)	474.07±659.23	192.56	
**pN stage** (n=373)				
N0	248 (66.5)	269.68±348.71^f^	148.70	0.001^a^
N1	45 (12.1)	542.26±1165.68^g^	103.29	
N2	73 (19.6)	542.69±747.67^fg^	285.50	
N3	7 (1.9)	327.96±195.51	270.51	
**pM stage** (n=301)				
M0	291 (96.7)	323.33±617.06	149.64	0.049^c^
M1	10 (3.3)	772.06±1092.70	295.49	
**Sex**				
Male	275 (73.5)	379.57±673.67	172.52	0.477^b^
Female	99 (26.5)	327.22±474.65	178.87	

^d^p=0.009, ^e^p=0.026, ^f^p=<0.001, ^g^p=0.019 ^a^p-values were estimated by Kruskal-Wallis 1-way ANOVA test. ^b^p-value were estimated by student's T test. ^c^p-value were estimated by Mann-Whitney U test.

**Table 2:** Univariate and multivariate Cox's regression analysis of gene expression for overall survival of patients with bladder cancer.

Characteristic	No. (%)	OS				
		CHR (95% CI)	P-value		AHR (95% CI)	P-value
SUSD2						
Low	258 (69.0)	1.00			1.00	
High	116 (31.0)	1.70 (1.13-2.56)	0.010		1.53 (1.01-2.31)	0.043

Abbreviation: DSS, disease**-**specific survival; DFS, disease**-**free survival; CHR, crude hazard ratio; AHR, adjusted hazard ratio AHR were adjusted for AJCC pathological stage (II, III and IV VS. I).

**Table 3:** Correlation between SUSD2 expression and clinicopathological characteristics of patients with bladder cancer.

			SUSD2						
			Low (≤65)			High (>65)			
Variables	Item	Patient No. (%)	No	%		No.	%		*p* value*
		189 (100.0)	93	49.2		96	50.8		
Age (y)	≤60	86 (45.5)	43	46.2		43	44.8		0.842
	>60	103 (54.5)	50	53.8		53	55.2		
Sex	Female	38 (18.3)	17	18.3		21	21.9		0.538
	Male	151 (81.7)	76	81.7		75	78.1		
Grade	I	63 (33.9)	36	39.1		27	28.7		0.161
	II	81 (43.5)	40	43.5		41	43.6		
	III	42 (22.6)	16	17.4		26	27.7		
Stage	I/II	153 (81.0)	86	92.5		67	69.8		<0.0001
	III/IV	36 (19.0)	7	7.5		29	30.2		
Pathology	Urothelial carcinoma	160 (84.7)	79	84.9		81	84.4		0.002
	Squamous cell carcinoma	16 (8.5)	3	3.2		13	13.5		
	Adenocarcinoma	13 (6.9)	11	11.8		2	2.1		
